# Noble Metal Particles Confined in Zeolites: Synthesis, Characterization, and Applications

**DOI:** 10.1002/advs.201900299

**Published:** 2019-07-18

**Authors:** Yuchao Chai, Weixiang Shang, Weijie Li, Guangjun Wu, Weili Dai, Naijia Guan, Landong Li

**Affiliations:** ^1^ School of Materials Science and Engineering National Institute for Advanced Materials Nankai University Tianjin 300350 China; ^2^ Key Laboratory of Advanced Energy Materials Chemistry of Ministry of Education Collaborative Innovation Center of Chemical Science and Engineering Nankai University Tianjin 300071 China

**Keywords:** catalysis, nanoparticles, Noble metals, optics, zeolites

## Abstract

Noble metal nanoparticles or subnanometric particles confined in zeolites, that is, metal@zeolite, represent an important type of functional materials with typical core–shell structure. This type of material is known for decades and recently became a research hotspot due to their emerging applications in various fields. Remarkable achievements are made dealing with the synthesis, characterization, and applications of noble metal particles confined in zeolites. Here, the most representative research progress in metal@zeolites is briefly reviewed, aiming to boost further research on this topic. For the synthesis of metal@zeolites, various strategies, such as direct synthesis from inorganic or ligand‐assisted noble metal precursors, multistep postsynthesis encapsulation and ion‐exchange followed by reduction, are introduced and compared. For the characterization of metal@zeolites, several most useful techniques, such as electron microscopy, X‐ray based spectroscopy, infrared and fluorescence emission spectroscopy, are recommended to check the successful confinement of noble metal particles in zeolite matrix and their unique physiochemical properties. For the applications of metal@zeolites, catalysis and optics are involved with an emphasis on catalytic applications including the size‐dependent catalytic properties, the sintering‐resistance properties, the substrate shape‐selective catalysis, and catalysis modulation by zeolite microenvironment.

## 1 Introduction

Metal particles dispersed on supports represent a simple but important type of functional materials, with large‐scale applications in some key industrial processes.^1^, ^2^, ^3^, ^4^, ^5^, ^6^, ^7^ Generally, the metal particles, according to their sizes, can be distinguished as the bulk particles (>100 nm), nanoparticles (1–100 nm), subnanometric particles (<1 nm), and even isolated atoms. Sometimes, the expression of clusters is used to describe the well‐defined metal particles with uniform size of below 2 nm. The particle size is known as a vital factor determining the performance of metal particles. Reducing the particle size results in an increase of the unsaturated coordination environment and the surface free energy of the metal species, making the metal sites more actively interacting with supports and adsorbates.^8^, ^9^ However, nanoparticles and subnanometric particles undergo aggregations easily and also suffer from sintering under the reaction conditions employed, which is frequently encountered with most of the typical supports, such as oxides^10^, ^11^ and carbon.^12^ In this context, to preserve the unique properties of nanoparticles and subnanometric particles for various applications, it is urgently desired to explore distinct types of supports to control the size and dispersion of metal particles by protecting them with interconnected pores and/or cavities. Zeolites, with characteristic micropores, uniform cage structures, and fine‐tuned acid–base sites, appear to be most promising host materials to confine the small metal particles, especially with size of <2 nm.^13^, ^14^ On the other hand, the micropores of zeolite hosts will bring about problems in the molecule diffusion and metal site accessibility, and the applications of metal particles confined in zeolite hosts are significantly restricted if the molecule diffusion has to be considered. The problem might be solved, at least partially solved, by using mesoporous zeolites with improved diffusion anility as host materials for metal particles.^15^, ^16^

**Figure 1:**
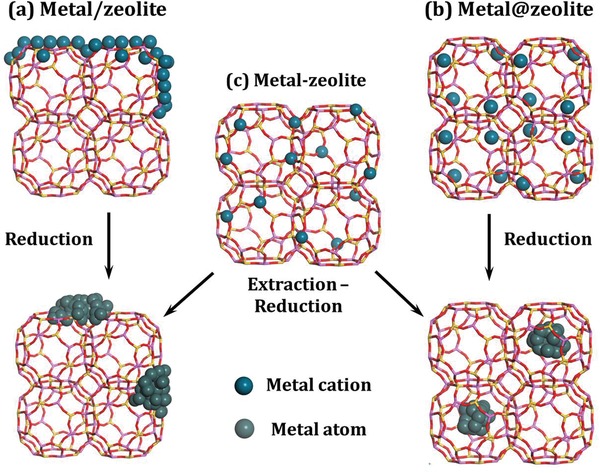
Three typical types of metal‐containing zeolites.

Since the success of large‐scale industrial production, zeolites have been widely used as catalysts and support materials.^17^, ^18^ Strictly speaking, zeolites are microporous aluminosilicates built by SiO_4_ and AlO_4_ tetrahedra linked into corner‐sharing networks, where the Si atoms bear charges of +4 and the Al atoms charges of +3.^19^, ^20^ The Si and/or Al atoms can be substituted by other elements to construct zeolite‐like materials such as phosphoaluminates. Due to the presence of aluminum in zeolite framework, electroneutrality requires the incorporation of charge compensating cations, which occupy extra‐framework sites in the channels and cages. For zeolites supported metal species, three typical types can be recognized: (a) metal species loading on the outer surface of zeolite crystals, (b) metal species encapsulated within the channels or cavities of zeolites, and (c) metal species incorporated into zeolite framework (**Figure** **1**). The samples of type (a), denoted as metal/zeolites, are usually prepared from zeolite supports and metal precursors via simple impregnation.^21^ The migration of metal species and their aggregation into larger particles always occur during the calcination and reduction processes. In contrast, for the samples of (b) denoted as metal@zeolites, the metal species are efficiently protected by the zeolite frameworks.^22^ The complex system of channels and micropores can provide strong confinement effects and significantly inhibit the particle growth to a particular size region. Meanwhile, the interconnect channels of zeolites allow the free access of guest molecules to the metal species confined in zeolites. Furthermore, with the strong confinement effects and the close proximity in the very limited spaces, cooperative bifunctional samples can be created in metal@zeolites from the metal particles and the intrinsic functional sites from zeolites, which are expected to find their wider use.^2^ While for the samples of (c) denoted as Me‐zeolites, metal species are incorporated into zeolite framework in the form of cations and further extraction and reduction processes are necessary to obtain zeolite supported metal particles.^23^, ^24^ In this context, the samples of (c) can be transformed to (a) or (b), depending on the detailed procedures of extraction and reduction.

In the past decades, impressive accomplishments dealing with the synthesis and applications of metal particles confined in zeolites have been achieved. Most recently, several elegant review papers dealing with different aspects of this important topic have been disclosed.^25^, ^26^, ^27^, ^28^, ^29^ Herein, we aim to present a summary on recent progresses on this topic and discuss some debatable questions involved. We hope that such a review article is useful for people working in related fields and is timely to boost further researches. For this purpose, we restrict our considerations on the frequently used noble metal particles, that is, Ru, Rh, Pd, Ag, Ir, Pt, and Au, confined in zeolites because of (i) the distinct properties of these noble metals with active outer‐shell electrons,^30^, ^31^ (ii) the great challenges of confining noble metal particles in zeolites due to their relatively low thermal stability and serious sintering upon treatments, and (iii) the widespread applications of zeolite‐confined noble metal particles. The most representative achievements on the synthesis and characterization of noble metal particles confined in zeolites will be focused. The unique catalytic properties of these samples in various important reactions will be discussed in details and their optics applications will also be included.

## 2 Synthesis Strategy to Noble Metal Particles Confined in Zeolites

Zeolites are usually synthesized by a process of slow crystallization of a silica‐alumina gel in alkaline or fluoride media at elevated temperatures. That is, a hydrothermal synthesis after a sol‐gel processing. In general, changing the synthesis conditions, such as the pH values of the gel, crystallization temperatures, and types of organic structure directing agents (OSDAs), might show significant impacts on the crystallization process and direct toward zeolites with different framework topologies.^30^, ^31^ On the other hand, the preparation of noble metal particles requires the strict control of experimental conditions since the high pH values and/or temperature will generally lead to the aggregation of particles.^32^, ^33^ Thus, it is quite necessary to explore the feasible combination between the synthesis of zeolite crystals and the regulation of noble metal particles, especially in the case of direct synthesis strategy.

### 2.1 Direct Synthesis from Inorganic Noble Metal Precursors

The direct or one‐pot synthesis of metal@zeolites offers attractive advantages over the multistep postsynthesis route. Most visibly, reducing synthesis steps can effectively reduce the production cost, making the direct synthesis route most attractive for industrial manufacture.^34^ Since the year of 2000, great efforts have been made on the one‐pot synthesis of metal@zeolites with specific functionalities and unique properties.^35^ Adding the inorganic noble metal precursors to the synthesis gels of zeolites might set the oxides of noble metals within zeolite crystals after crystallization process, deriving metal@zeolites upon reduction procedures.

Ru or RuO_2_ nanoparticles have been disclosed as unique candidates to be confined in zeolite crystals via one‐pot synthesis route due to their good stability. White et al.^36^ and Weitkamp and co‐workers^37^ reported the one‐pot synthesis of entrapped RuO_2_ clusters in the void volume of large pore and medium pore zeolites in the absence of OSDAs, respectively. Typically, White et al. synthesized RuO_2_ nanoclusters in the supercages of FAU zeolite by one‐step hydrothermal method and the size of RuO_2_ clusters was defined to be ≈1.3 nm by the space of supercages.^36^ In a similar way, Weitkamp and co‐workers synthesized subnanometric RuO_2_ particles entrapped in the MFI zeolite starting from simple metal salts, RuCl_3_.^37^ The RuO_2_ particles with size of 0.5–0.9 nm were primarily located inside the pores of the MFI zeolite. The direct synthesis of entrapped RuO_2_ clusters was further extended to the small pore zeolite LTA, which was most important and challenging since the small pore size impeded the use of common postsynthesis strategies. Zhan and Iglesia succeeded in encapsulating RuO_2_ clusters with size of ≈1 nm in LTA cages.^38^ The encapsulated RuO_2_ clusters could undergo easy reduction to Ru clusters at below 393 K and Ru@LTA was obtained.

It should be emphasized that the one‐pot synthesis of metal@zeolites or MeO*_x_*@zeolites from inorganic noble metal precursors is very difficult primarily due to the fast precipitation of noble metal oxides under the alkaline conditions of zeolite synthesis. The unique tolerance of ruthenium ions to alkaline conditions at elevated temperatures leads the success in the direct synthesis of RuO_2_@zeolites. Unfortunately, other noble metal oxides cannot be encapsulated in zeolites via similar routes, and modifications or improvements are therefore proposed for the direct synthesis strategy.

### 2.2 Direct Synthesis from Ligand‐Assisted Noble Metal Precursors

In the past decade, the ligand‐assisted noble metal precursors, as the most straightforward strategy to stabilize metal cations in the form of organometallic complexes, have been explored and employed in the direct synthesis of metal@zeolites. The high alkaline conditions (pH > 12) required for hydrothermal crystallization of zeolites typically cause the precipitation of inorganic precursors as colloidal metal hydroxides larger than the zeolite voids, thus preventing their encapsulation. Organic amines can effectively stabilize metal cations and prevent their premature precipitation. On the other hand, organic amines can act as coordinating agents to encourage the sequestering of precursors during the incipient formation of aluminosilicate frameworks.^39^ Iglesia and co‐workers have made remarkable contributions to this protocol, and a series of noble metal particles have been successfully encapsulated in the cavities of zeolites, such as (Rh, Pd, Ag, Pt, Au)@LTA^40^ and (Ru, Rh, Pd, Pt)@(SOD, ANA, GIS).^41^ Whether the ligand‐assisted metal precursors are eligible for the direct synthesis of metal@zeolites depends on the stability of precursors under the typical conditions of zeolite synthesis. Indeed, this protocol describes a self‐assembling strategy without the use of OSDAs, like the ship‐in‐a‐bottle synthesis.^42^ Hydrothermal synthesis of these zeolites proceeds via three main steps: induction, nucleation, and crystallization. The framework building units are denoted as the “bottle” and the external component (noble metal complexes) as the “ship.” Zeolite nucleation and crystallization hinge on a balance of ship‐bottle assembly with the surrounding zeolite framework stabilized by the central metal complexes. The cationic noble metal complexes used here are hydrophilic and are effective in templating Al‐rich zeolites during the hydrothermal synthesis owing to the charge matching between (noble metal complexes)*^n^* ^+^ and (Al‐rich zeolite framework)*^n^* ^−^. These materials are typically prepared without OSDAs at low crystallization temperatures (353–373 K), and accordingly, only zeolites with Al‐rich frameworks (Si/Al < 3) have been achieved.

While the above‐mentioned results are very interesting, the low‐silica zeolites suffer from low hydrothermal stability, which significantly limits their applications as industrial catalysts. In this context, the synthesis of noble metal particles encapsulated in high‐silica or pure‐silica zeolites should be of both fundamental and practical significance. High‐silica or pure‐silica zeolites are usually synthesized in the presence of OSDAs, at higher crystallization temperatures (>423 K) and under higher alkalinity. Under such conditions, most noble metal precursors tend to precipitate as bulk metal hydroxides.^33^ Therefore, it is necessary to search for the more effective ligands to stabilize the noble metal cations during the synthesis of Si‐rich zeolites. Several years ago, it was reported that mercaptoalkyl trimethoxysilane ligands could simultaneously interact with metal clusters through the mercapto groups and with silica precursor (TEOS) through the alkoxysilane groups, leading to stable metal clusters encapsulated in silica via proper post‐treatment procedures.^43^

**Figure 2:**
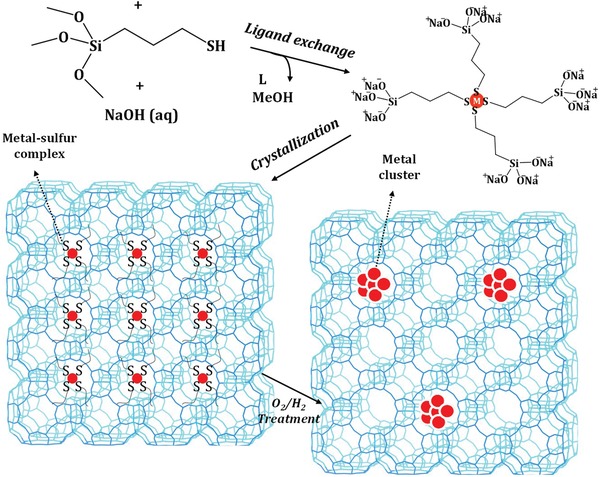
Schematic process for mercaptosilane‐assisted metal encapsulation during zeolite crystallization. Adapted with permission.^45^ Copyright 2010, American Chemical Society.

Iglesia and co‐workers developed a similar strategy to encapsulating small (1–2 nm) and nearly monodisperse Au clusters in LTA or MFI zeolites using mercapto‐assisted hydrothermal synthesis protocol.^44^ The mercaptosilane ligands could protect Au^3+^ cations against reduction when added into zeolite synthesis gels. Post‐treatment of the crystallized zeolites in O_2_ and then H_2_ could reduce the Au^3+^ cations to encapsulated Au clusters, that is, Au@LTA and Au@MFI. Explicitly, the strong bonding of the mercapto group to metal species and the copolymerization of the alkoxysilane group with the zeolite organosilane precursor ensure the efficient encapsulation of highly dispersed metal centers within zeolite matrix. In a step forward, this kind of ligands is widely used in the synthesis of other noble metal particles encapsulated in zeolites. For example, Iglesia and co‐workers reported the encapsulation of noble metal clusters during the hydrothermal crystallization of LTA zeolite using bifunctional (3‐mercaptopropyl)trimethoxysilane ligands (**Figure** **2**).^45^ The successful encapsulation of Pt, Pd, Ir, Rh, and Ag clusters within the LTA zeolite was accomplished. Furthermore, Corma and co‐workers presented a direct synthesis strategy of a nanocrystalline high‐silica CHA zeolite with encapsulated Pt particles of ≈1 nm (Pt@CHA), using the combination of N,N,N‐trimethyl‐1‐adamantyl ammonium hydroxide and Pt‐mercapto complex as OSDA and Pt precursor, respectively.^33^ The encapsulated Pt particles were kept unchanged in H_2_ and steam, while they fragmented into stable site‐isolated Pt atoms in O_2_ at 923 K, building a fully reversible cluster formation/fragmentation cycle. The mercapto ligands usually have poor solubility in water and it takes a long time to obtain the homogeneous mixture of metal‐mercapto precursor and SiO_2_ colloid, resulting in the poor reproducibility of this synthesis route. Very recently, Li and co‐workers improved the strategy to zeolite‐encapsulated Pd nanoparticles with the mixed‐solvent strategy during the preparation of ligand‐stabilized Pd precursor.^46^ Briefly, Pd‐mercapto precursor dissolved well in alcohol‐water mixed solvent to form clear solution and homogeneous gel was formed through the pre‐hydrolysis process only in 6 h. After hydrothermal crystallization and conventional treatment, Pd nanoparticles with apparent size of about 1.8 nm were encapsulated in the channels of MFI zeolites, that is, Pd@MFI.

In addition to the mercapto groups, other organic ligands, such as polyvinyl pyrrolidone (PVP)^47^, ^48^ and 3‐aminopropyltrimethoxysilane (APTMS),^49^ were also used to stabilize noble metal cations at high crystallization temperatures and under high alkaline conditions. Although noble metal particles could be confined inside zeolite crystals, the size of metal particles appeared to be slightly larger (several nanometers). By using ethidene diamine as stabilizing ligands and tetrapropylammonium hydroxide (TPAOH) as OSDAs, Yu and co‐workers developed an interesting strategy to encapsulating ultrasmall Pd clusters within nanosized silicalite‐1 zeolites via direct hydrothermal synthesis.^50^

Actually, apart from some cases of noble metal nanoparticles confined in zeolites, for example, LTA, SOD, GIS, and ANA, through self‐assembling in the absence of OSDAs, the preparation of zeolites with higher Si/Al ratios always involves the use of OSDAs (usually are quaternary ammonium salts, QASs).^30^ These OSDAs organic cations are much bulkier than alkaline inorganic cations, and consequently, the total positive charges introduced into the hybrid inorganic–organic matrix are lower, requiring less aluminum atoms in the framework. The structure and the electronic properties of the metal–ligand complexes are somewhat similar to the OSDAs, and the non‐noble metal–ligand complexes can be used as the sole templates to synthesize the Si‐rich zeolites in the absence of QASs.^51^ However, to the best of our knowledge, there are no reports on using noble metal–ligand complexes as the sole templates in the synthesis of Si‐rich zeolites, probably due to the inferior hydrothermal stability of noble metal–ligands complexes. In this context, the rational cooperation between the noble metal–ligands complexes and OSDAs plays an essential role in the successful synthesis of Si‐rich zeolites. That is, the noble metal–ligands complexes should not be interfered with the template functions of OSDAs.

### 2.3 Multistep Postsynthesis Encapsulation

Both the stability of noble metal precursors and the precise combination between precursors and OSDAs are intractable problems in the direct hydrothermal synthesis of metal@zeolites, which might be bypassed via the multistep postsynthesis strategy. If it is difficult to find suitable organic ligands to stabilize the noble metal precursors, the interzeolite framework transformation under mild conditions becomes an alternative choice. Actually, interzeolite transformation provides a general route for the encapsulation of noble metal particles in microporous solids, in which the successful placing of precursors within a parent zeolite structure can be accomplished via hydrothermal crystallization. The parent structure, containing metal particles within its microporous voids, can then be recrystallized without loss of encapsulation into a daughter structure (the target zeolite) of higher framework density.^41^, ^52^ For example, MFI is a medium‐pore Si‐rich zeolite that typically requires high crystallization temperatures (423–473 K) and high alkaline conditions (pH > 11) for its hydrothermal synthesis, and the encapsulation of noble metal particles remains challenging by ligand‐stabilized metal precursors. Iglesia and co‐workers developed a general strategy for the encapsulation of noble metal clusters (Pt, Ru, Rh) within MFI by exploiting interzeolite transformation of BEA or FAU zeolites (parent structure) into MFI zeolite (daughter structure) via low‐temperature hydrothermal treatment.^32^

**Figure 3:**
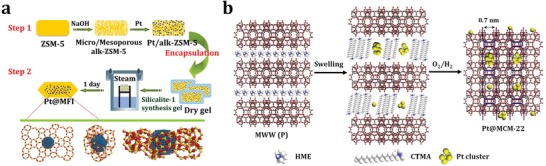
a) Schematic of the two‐step dry‐gel‐conversion synthesis of Pt nanoparticles encapsulated in MFI zeolite. Adapted with permission.^53^ Copyright 2015, American Chemical Society. b) Schematic of confining Pt subnanometric particles in MWW zeolite during interzeolite transformation. Adapted with permission.^54^ Copyright 2017, Nature Publishing Group.

In contrast to the one‐pot hydrothermal route where the materials are in direct contact with water, the daughter gel in the multistep postsynthesis route might contact with steam or mixed vapors of steam and organic structure directing agents, which is beneficial for synthesis. Ding and co‐workers developed an effective two‐step dry‐gel‐conversion synthesis route to Pt@MFI (**Figure** **3**a).^53^ In the first step, alkali treatment was employed to introduce intracrystalline mesopores within commercial ZSM‐5 zeolites, after which Pt nanoparticles were introduced by the impregnation to derive Pt‐containing hierarchical ZSM‐5. The mesopores in such hierarchically structured zeolites had high accessibility and transportability, resulting in the distribution of Pt nanoparticles inside zeolite crystals rather than on the outer surface. In the second step, the Pt‐containing ZSM‐5 was dispersed in the synthesis gel of silicalite‐1, which not only filled the large voids of hierarchical ZSM‐5 but also formed a silica coating to further encapsulate the Pt nanoparticles in zeolite crystals. Finally, Pt@MFI with different Pt contents could be obtained by steam‐assisted crystallization. Recrystallization of the zeolite in the presence of noble metal precursors and related techniques present some promising interzeolite‐transformation approaches to encapsulate noble metal nanoparticles and subnanometric particles inside zeolite crystals. Recently, Corma and coo‐workers developed a process of MWW‐interzeolite transformation to confine Pt subnanometric particles, which was not realized by direct hydrothermal synthesis.^54^, ^55^ 2D MWW layers were first prepared via traditional hydrothermal route in the presence of OSDAs and subsequently the dispersion of sub‐nanometer Pt particles in DMF was added. Once the OSDAs were removed upon calcination, the 2D MWW layers transformed into 3D MWW structure with Pt species encapsulated inside the pores (Figure 3b).

Hollow zeolites represent an emerging type of structured zeolite materials with improved diffusion properties, which can be used as nanocontainers to confine and stabilize the well‐defined noble metal nanoparticles.^56^ Farrusseng and co‐workers developed a strategy to prepare Pt and Au nanoparticles confined in hollow ZSM‐5 and silicalite‐1 crystals by impregnation of parent microporous zeolite crystals with the solution of a metal salt, followed by desilication‐recrystallization with TPAOH solution, calcination, and reduction.^57^ The resulting yolk–shell materials were defined by controllable metal particle size distributions, the complete confinement of noble metal nanoparticles inside the zeolite shell, and, accordingly, the high resistance to sintering during the high‐temperature reductive treatment. In a step forward, this strategy was extended to the encapsulation of bimetallic alloy nanoparticles, for example, AuAg, PdAg, PtAg, and PdPt, inside the zeolites crystals.^58^

OSDA‐free synthesis of zeolites is highly desired since those OSDAs always contribute to the most significant production costs of zeolites and the calcination removal of OSDAs will further cause environmental problems. In recent years, the OSDA‐free synthesis of microporous materials has renewed its interest thanks to the direct preparation of zeolites with higher Si/Al ratios by the seeding procedures.^59^ Xiao and co‐workers developed the seed‐directing methodology for the encapsulation of noble metal nanoparticles in zeolites.^60^, ^61^ The key for the preparation of Pt@BEA and Pd@BEA samples was the use of zeolite seeds containing metal nanoparticles from impregnation, which were covered by a crystalline zeolite sheath after the hydrothermal synthesis. This strategy can work without the use of organic stabilizers and lead to metal nanoparticles of <4 nm.

These above‐mentioned postsynthesis strategies can lead to the successful confinements of noble metal nanoparticles and subnanometric particles within zeolite crystals, which are sometimes not feasible via direct hydrothermal synthesis. It is impossible to compare the advantages and disadvantages of these two types of strategies, since the results are highly dependent on detailed experiment parameters. Nevertheless, it can be seen that most direct synthesis routes lead to the encapsulation of metal particles within zeolite channels or cavities while postsynthesis routes do not.

### 2.4 Ion‐Exchange Followed by Reduction

For the synthesis of strictly well‐defined noble metal nanoparticles, that is, clusters, confined in zeolites, more sophisticated techniques should be developed. Ion‐exchange is historically one of the first means of introducing the metal sites into the zeolites. Traditionally, ion‐exchange is performed in an aqueous medium using successive cycles of treatment in an excess of targeted cations and washing. It is easy to control the number of the exchanged metal sites; however, the exchanged metal sites may locate both inside zeolite channels and on the outer surface. In this context, the post‐treatment procedures that drive the noble cations to the zeolite cavities are most important to obtain the expected metal@zeolites. The preparations of noble metal clusters from ion‐exchanged zeolite Y were reported many years ago and the detailed processes usually included the oxidation, decomposition, and reduction of the noble metal precursors.^24^, ^62^ To prepare well‐dispersed platinum particles in FAU zeolite, the use of Pt(NH_3_)_4_^2+^ as precursor for ion‐exchange was recommended, and very small Pt particles with 13–20 atoms per particle and of an average size below 1.1 nm were prepared in NaY and H‐USY from ion‐exchanged followed by reduction strategy.^63^ Xu and co‐workers developed the similar strategy for the encapsulation of gold nanoclusters of 1 nm within the supercages of HY zeolite.^64^ Some zeolites, such as MCM‐22, ferrierite, and sodalite, can be obtained from their layered precursors MCM‐22P, PREFER, and RUB‐15, respectively.^65^ These layered precursors with flexible layer distance are good candidates for the introduction of the guest noble metal precursors or nanoparticles. Very recently, subnanometric Pd clusters with 1.4 wt% loading were prepared via the ion‐exchange of positively charged cetyltrimethylammonium CTA^+^ with Pd precursors during the transformation of FER layers into FER zeolite.^66^ More simply, the PdO@MOR zeolite with high Pd dispersion was directly prepared via exchanging commercial MOR zeolite with certain amount of Pd(NO_3_)_2_ solution.^67^ However, the exact location of Pd species remained unknown. The agglomeration and leaching of noble metal ions are the key challenges for introducing them into zeolites. To overcome these bottlenecks, Román‐Leshkov and co‐workers developed a new strategy to bimetallic nanoparticles encapsulated within zeolite pores.^68^ Zn‐containing MFI zeolite (Zn‐MFI) was prepared via direct hydrothermal synthesis, and Pt^2+^ ions were exchanged into Zn‐MFI to produce Pt^2+^/Zn‐MFI, taking the advantage of charge imbalance generated by the Zn in the framework. After reduction treatment, composition‐specific PtZn*x* bimetallic nanoclusters were successfully encapsulated in the pores of the MFI zeolite, that is, PtZn*x*@MFI.

**Figure 4:**
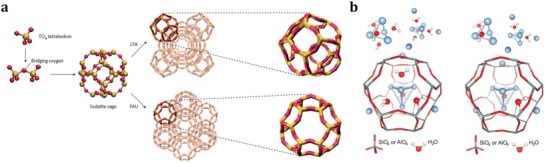
a) Schematic of LTA and FAU zeolite framework and their building blocks. Adapted with permission.^73^ Copyright 2016, Nature Publishing Group. b) Structures of Ag_4_(H_2_O)_4_ and Ag_4_(H_2_O)_2_ embedded in the sodalite cage. Adapted with permission.^74^ Copyright 2018, American Association for the Advanced of Science.

LTA or FAU‐type zeolites exchanged with silver ions become fluorescent after calcination, due to the formation of small clusters, such as Ag_3_ *^n^* ^+^ and Ag_6_ *^m^* ^+^, in the zeolite cages via an autoreduction process.^69^, ^70^ LTA and FAU zeolites are unique hosts for silver clusters due to their easy exchange with Ag^+^ ions and well‐defined crystal structures with cages and channels of molecular dimensions. In fact, Ag@LTA and Ag@FAU materials, with discrete electronic energy, are the most widespread applications of confining noble metal clusters in zeolites via ion‐exchange followed by reduction.^71^, ^72^ As shown in **Figure** **4**a, both FAU and LTA zeolites contain sodalite cages, but differ in their interconnecting secondary building units; a double six‐ring for FAU and a double four‐ring for LTA.^73^ The controllable loading of Ag clusters was achieved by exchanging these zeolites with different amounts of Ag precursors, yielding fully or partially exchanged FAU and LTA zeolites. Autoreduction processes during calcination of zeolites led to the formation of Ag clusters, with the required electrons originating from the expulsion of oxygen atoms and/or the oxidation of hydration water to oxygen. Moreover, the formation of bright emitting Ag clusters could be induced by a photochemical reduction of the exchanged Ag^+^ using focused light irradiation. The confined Ag clusters were generally small in size, containing less than ten atoms, and could be positively charged, which were, for example, determined to be a mixture of tetrahedral Ag_4_(H_2_O)*_x_* ^2+^ (*x* = 2 and *x* = 4) clusters in the sodalite cages (Figure 4b).^74^ The concrete applications of Ag@LTA or FAU will be described in Section 3 in details. Briefly, because of their unique spectral features such as the large Stokes shift and the high external quantum efficiency, these materials could be potentially used as phosphors for the fabrication of fluorescent lamps and as wavelength convertors in solar cells.^75^

Overall, favored by its simplicity and economic advantages, ion‐exchange with solutions of metal salts is the most straightforward and the most frequently applied means to introduce noble metal centers into the zeolite crystals. In the absence of specific metal–zeolite interactions and uniform charge densities, however, ion‐exchange can lead to an uncontrollable distribution of the noble metal sites both inside the pores and on the outer surface upon reduction post treatments. So far, Ag@LTA and Ag@FAU are established as elegant examples of confining Ag clusters in zeolites cages via ion‐exchange followed by reduction.

## 3 Characterization of Noble Metal Particles Confined in Zeolites

Reliable characterization techniques are required to reveal the successful confinements of noble metal particles in zeolite crystals as well as their specific properties. Notably, the specific microenvironment of noble metal particles confined in zeolites, as an important type of the strong metal–support interaction materials, is very complicated. Therefore, characterization of dispersed noble metal particles confined in zeolites is a hard task. In **Table** **1**, we summarize the frequently used techniques for the characterization of noble metal particles confined in zeolites and the representative references are also included. In most cases, to fully understand the structural features and functionalities of noble metal particles confined in zeolites, a combination of several techniques should be applied. For instance, Gates and co‐workers developed a strategy for the characterization of ultradispersed Ir particles confined in FAU zeolites via the combination of aberration‐corrected high‐angle annular dark‐field scanning transmission electron microscopy (HAADF‐STEM), extended X‐ray absorption fine structure (EXAFS) spectroscopy, and Fourier transform infrared spectroscopy (FTIR) with CO as probe molecules.^83^ Furthermore, with the rapid development of operando techniques in the past two decades, we can now characterize the dynamic structure of noble metal particles confined in zeolites as well as their interaction with zeolite framework. In this section, we will discuss the essential techniques to confirm the successful confinement of noble metal particles in zeolites and their structural characteristics.

**Table 1:** Characterization techniques for noble metal particles confined in zeolites

Characterization technique	Information obtained	Ref.
Temperature‐programmed techniques (TPD, TPR, TPO, TPSR)	Structure, stability, and reactivity of metal centers	46, 47
Computational modeling	Structure and reactivity of metal centers	46, 76
Electron microscopy (TEM, STEM)	Structure and distribution of metal centers	54, 77
Nuclear magnetic resonance spectroscopy (NMR)	Framework structure and acidity/basicity of zeolite hosts	46, 78
Gas physisorption	Textural properties of zeolite hosts	47, 79
Atom probe tomography	Spatial distribution of elements	80
UV–vis spectroscopy	Structure and electronic states of metal centers	56, 81
Vibrational spectroscopy (FTIR, Raman)	Structure of metal centers; acidity/basicity of zeolite hosts	46, 52
X‐ray absorption/emission spectroscopy (XAS/XES) X‐ray photoelectron spectroscopy	Structure, electronic state, coordination state of metal centers	56, 82
X‐ray diffraction (XRD), small‐angle X‐ray scattering (SAXS)	Structure, distribution, and geometry of metal centers; crystal phase purity of zeolite hosts	46, 47

### 3.1 Electron Microscopy

**Figure 5:**
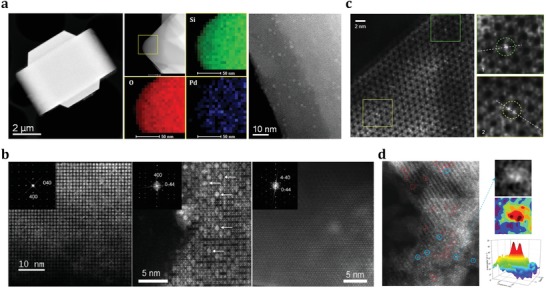
a) HAADF‐STEM image, selected‐area element mapping analyses, and high‐resolution HAADF‐STEM image of Pd@MFI sample. Adapted with permission.^46^ Copyright 2018, American Chemical Society. b) Aberration‐corrected STEM‐HAADF micrographs of dehydrated Ag@LTA recorded along [001], [011], and [111] directions. Adapted with permission.^87^ Copyright 2011, Wiley‐VCH. c) Aberration‐corrected HAADF‐STEM image of Pt@MWW, where two zoom‐in are shown in the square regions. Adapted with permission.^54^ Copyright 2017, Nature Publishing Group. d) Aberration‐corrected HAADF‐STEM images characterizing Rh@FAU with intensity surface plot and 3D intensity surface plot. Adapted with permission.^88^ Copyright 2016, American Chemical Society.

Naturally, the most intuitive demonstrations of noble metal particles confined in zeolites are seeing them directly, that is, metal particles being separated from each other and located at specific positions within zeolite supports. Electron microscopy is always the first choice for the characterization of metal@zeolites. The reconstructed tomogram and 3D TEM tomography are most desired to confirm if the noble metal particles are confined or embedded within the zeolite crystals or located on the external surface of zeolite crystals.^43^, ^61^ Unfortunately, such techniques are costly and not easily available. The stability of noble metal particles in zeolites can be investigated by ex situ or in situ TEM, where confined nanoparticles within the zeolite crystals are highly stable against sintering while nanoparticles located on the outer surface undergo serious sintering upon calcination and/or reduction. While TEM is a routine technique for studying typical noble metal nanoparticles confined in zeolite, the resolution of TEM is, in most cases, insufficient for the characterization of ultrasmall particles below 2 nm.^84^ For the reliable analysis of ultrasmall particles with atomic resolution, the HAADF‐STEM, based on scanning the specimen across with the electron beam, is preferred. In comparison with conventional TEM, this technique results in a stronger interaction of the electrons with the sample and, therefore, more types of signals with high spatial resolution can be produced.^85^ These signals include characteristic X‐rays that are useful for reconstructing the energy‐dispersive X‐ray (EDX) elemental maps and inelastically scattered electrons allowable for electron energy loss spectroscopy (EELS) analyses.^86^ The most important signals come from elastically backscattered electrons, which are used to reconstruct the dark‐field atomic resolution image of the sample, by applying an HAADF detector. The surge in interest in the subject of atomically dispersed metal@zeolites essentially coincided with the availability of STEM for observation of individual metal species on supports. For instance, using of the HAADF‐STEM method, Li and co‐workers clearly observed the uniform Pd nanoparticles dispersed in MFI zeolite crystals with apparent size of 1.7–1.8 nm, that is, the bright dots in the images (**Figure** **5**a).^46^ Aberration‐corrected HAADF‐STEM was employed to investigate the location of Ag clusters inside the LTA zeolite crystals.^87^ The intact and empty straight channels were clearly observed, indicating that most of the Ag clusters were located in the supercages of LTA zeolite (Figure 5b). In another work, HAADF‐STEM was employed to investigate the exact location of Pt in MWW zeolite crystals, where Pt single atoms and small clusters appeared to be confined in the cups and at the connecting walls between the cups while some tiny Pt clusters (≈0.7 nm) could also be encapsulated in the supercages of MWW zeolites (Figure 5c).^54^

Generally, the obtained STEM images are characterized by the Z‐contrast, since electron scattering efficiency depends on the element atomic number.^85^ This property is particularly good for studying atoms/clusters of noble metals (as the heavy elements) dispersed within the zeolite crystals composed of light elements Si, Al, and O. This concept was skillfully utilized in the study of Rh clusters confined inside the pores of FAU zeolite by Gates and co‐workers.^88^ Delicate HAADF‐STEM analyses of fresh Ru@FAU sample and those after different reductive treatments allowed to track the dimer intermediates during the sintering of the Rh species (Figure 5d). As shown above, HAADF‐STEM is a truly versatile tool for the analyses of metal@zeolites especially dealing with confined subnanometric metal particles. By our assessment, atomic‐resolution images are essential for establishing the atomic dispersion of noble metal in zeolite matrix. STEM images become most valuable when various parts of metal@zeolite samples are carefully characterized and statistical data on the size and distribution of noble metal particles are provided.^89^, ^90^ It should be noted that zeolites offer the advantages of structural uniformity for electron microscopic analyses, however the framework of zeolites, especially with low Si/Al ratios, are very sensitive to the electron beams.^91^ The sample damages under high‐energy electron beams that are necessary for taking high‐resolution images frequently occur during the measurements, which is expected to be solved with the further developments of electron microscopy.

### 3.2 X‐Ray Based Spectroscopy

**Figure 6:**
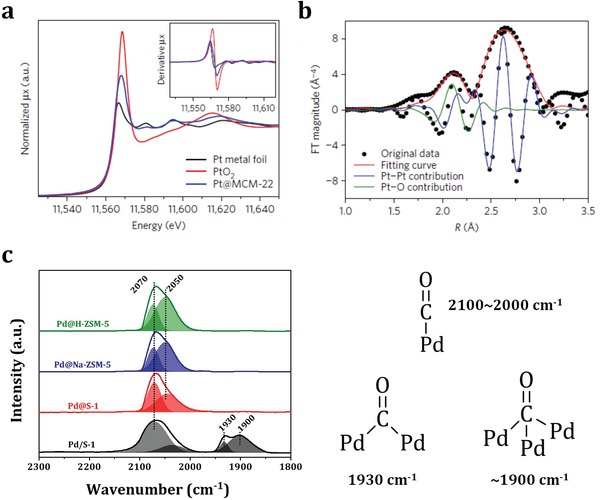
a) XANES spectrum of Pt@MWW with the first‐order derivative spectrum given in the inset; b) Fourier transform of k3‐weighted EXAFS spectrum of the Pt@MWW sample. Adapted with permission.^54^ Copyright 2017, American Chemical Society. c) FTIR spectra of CO adsorption on Pd/MFI and Pd@MFI with corresponding CO adsorption configurations. Adapted with permission.^46^ Copyright 2018, American Chemical Society.

X‐ray diffraction (XRD) and X‐ray photoelectron spectroscopy (XPS) have become routine laboratory tools in material characterization over the past decades. For metal@zeolites, XRD is commonly employed to determine the topology of zeolite hosts. Due to their high dispersion and low loadings in metal@zeolites, no diffraction signals corresponding to confined noble metal particles can be distinguished in the XRD patterns.^49^, ^50^ XPS is employed to determine the electronic states of confined noble metal species in zeolites. Compared to those supported on the outer surface of zeolite crystals, the noble metal particles confined in zeolite crystals give much lower peak intensities since XPS belongs to shallow surface analysis technique.^46^, ^92^ For characterizing the coordination states and environment of noble metal particles confined in zeolites, synchrotron‐based X‐ray techniques are highly desired. In this regard, X‐ray absorption (XAS) is the most commonly applied technique that can be even performed with in situ or operando modes. XAS cannot provide visualized evidences as those from electron microscopy, while XAS spectroscopy with multiple‐shell analyses is more informative. Briefly, XAS is an element‐selective technique recording the absorption spectra during core‐electron excitation, as a function of photon energy.^93^, ^94^ During XAS studies, two types of information are combined: X‐ray absorption near edge structure (XANES) and extended X‐ray absorption fine structure (EXAFS). XANES concerns the region right before 0.05–0.1 keV and after the absorption edge, providing information on the oxidation states of selected element and its local symmetry.^95^ Usually, XANES spectra of the studied samples are compared with the reference spectrum to fingerprint the spectral features with some known structural properties. In the system of Pt@MWW where most confined Pt species existed in the metallic states,^54^ the presence of Pt—O bonds in the sample was revealed by XANES spectrum. The curve shape looked like metallic Pt while the white line intensity was higher (**Figure** **6**a), indicating the presence of Pt—O bonds and the strong interaction between Pt and MWW zeolite. It is important to note that the quality of XANES results is hardly influenced by the thermal disorder in solids as a function of temperature and XANES spectra can be recorded extremely fast (as fast as tens of picoseconds).^96^, ^97^ All these make XANES an ideal choice for in situ or operando studies. In situ Ag‐K edge XANES spectra of reference and Ag@FAU samples were recorded with increasing temperatures. The energy changes in the first peak of pretreated samples clearly revealed the changes in oxidation states of Ag clusters confined in FAU zeolite.^98^

In turn, EXAFS deals with oscillations in the high energy part of the absorption spectra originated from the backscattering of the emitted low‐wavelength photoelectrons by neighboring atoms.^99^ Analyses of the oscillation patterns provide structural details, for example, chemical specificity, coordination numbers, and bonding distances, of several coordination shells around the noble metal particles. For example, Au clusters confined in FAU zeolites were successfully characterized by EXAFS.^100^ The detailed data about various bonds, including Au—Au, Au—O, and Au—Al, directly revealed the structure configurations of confined Au clusters. Indeed, using the entire sets of coordination numbers in conjunction with their uncertainties can provide more in‐depth information on the structure of confined metal particles than the commonly used size and morphology. For noble metal particles with diameters smaller than 3–5 nm, the coordination number shows strong and nonlinear dependence on the particle diameter.^101^ This property has been widely used in EXAFS analysis to determine the size of noble metal nanoparticles confined in zeolites. For example, according to the fitting results of the EXAFS spectrum (Figure 6b),^54^ the coordination number of Pt in Pt@MWW was determined to be 4.7, corresponding to Pt clusters with size of less than 0.7 nm (less than 13 atoms). Furthermore, if the shape‐sensitive modeling scheme proves reliable resemble the size modeling, the structure refinement could be attempted. This strategy may also provide a reliable estimation of the confined noble metal particles, which in turn can be directly compared with electron microscopy observations. Besides, potential techniques based on the emissions of core hole after X‐ray absorption, including X‐ray emission spectroscopy (XES), resonant XES, and resonant inelastic X‐ray scattering (RIXS), are developed.^102^, ^103^ These techniques may become highly sensitive tools for studying the detailed electronic structure of noble metal particles confined in zeolites.

### 3.3 Infrared and Fluorescence Emission Spectroscopy

Fourier transform infrared spectroscopy (FTIR) is a versatile tool for material characterization. The well‐known applications of vibrational spectroscopy are determining Brønsted and Lewis acid sites (BAS and LAS) in zeolites.^104^, ^105^ It can be performed either by directly observing the band at ≈3610 cm^−1^, related to bridging hydroxyl groups, that is, BAS, or by using probe molecules such as pyridine for quantifying acid sites inside the pores. On the other hand, FTIR spectroscopy with probe adsorption is also a powerful and widely applied technique for the characterization of supported noble metal species. Generally, terminal carbonyl bands characterize both mono‐ and oligonuclear metal centers, while bridging carbonyl bands are formed with neighboring metal centers.^106^ This technique is sensitive and most valuable in distinguishing subnanometric and larger particles. Very recently, Li and co‐workers studied the Pd particles confined in MFI zeolites by means of FTIR spectroscopy with CO adsorption (Figure 6c).^46^ For Pd@MFI, only atop‐bonded CO species (IR bands at 2100–2000 cm^−1^) were observed while doubly bridge‐bonded (IR bands at ≈1930 cm^−1^) and threefold bridge‐bonded CO species (IR bands at ≈1900 cm^−1^) were missing, indicating the formation of confined subnanometric Pd particles in MFI zeolites. Furthermore, CO can interact with noble metal sites by σ‐coordination, when the electrons from the lone pair of CO are transferred to the empty orbitals of a metal atom, or by back π‐donation from the partially filled d‐orbitals.^91^ The resulting red or blueshifts from the C—O bond frequency in the gas phase, depending on the strength and nature of corresponding interactions, are useful in studying of the properties of the noble metal particles. Two CO adsorption bands could be observed on Pt metal sites,^54^ due to CO interacting in lineal and bridge configurations, respectively. The noticeable shifts of these two bands compared to gas‐phase CO suggested that the zeolite matrix could act as an electron donor and increased the electron density of encapsulated Pt clusters. Moreover, in the FTIR spectra of CO adsorption on Rh@M^+^Y zeolites (M = H, Na, K, Rb, and Cs), it was clear that the vibration frequency of CO on Rh sites shifted toward lower wavenumbers with increasing cation radius, that is, H < Na < K < Rb < Cs, due to the changing interactions between Rh and zeolite matrix.^107^

It has been demonstrated that LTA or FAU‐type zeolites exchanged with Ag ions become fluorescent after calcination, due to the formation of small clusters, such as Ag_3_ *^n^* ^+^ an Ag_6_ *^m^* ^+^, in the zeolite cages via autoreduction processes. A red emitter with λ_em_ at 366 nm was typically formed in LTA‐type zeolites with different structures of intermediate Ag clusters.^71^ The color changes were attributed to the charge transfer between Ag and framework oxygen atoms. Thereupon, the fluorescence emission spectroscopy or imaging may be used to depict the electronic structures of noble metal clusters confined in zeolites. So far, this technique has been successfully employed and mostly limited in characterizing Ag clusters confined in LTA or FAU zeolites, where the reversible oxidation‐reduction of Ag clusters inside supercages could be achieved. According to our assessment, with the creation of noble metal clusters inside the supercages of zeolites, the fluorescence emission technique might find more broader applications, like the case of Pt@MWW sample.^54^

## 4 Miscellaneous Applications of Confined Noble Metal Particles

Noble metal particles confined in zeolites, that is, metal@zeolites, have been widely applied in many fields, and in fact, the synthesis and characterization of Metal@zeolites are driven by their applications. To summarize the applications of noble metal particles confined in zeolites, we first discuss the specific behaviors of isolated noble metal particles, which are known as key functional sites in the metal@zeolites. It is well known that noble metal particles with different sizes, for example, nanoparticles, subnanometric particles, and single atoms, can exhibit quite different behaviors in various processes.^25^ Many other factors, for example, the crystal shape and metal–support interaction, also show significant impacts on the physicochemical properties of noble metal particles and accordingly influence their specific behaviors. In this review article, we will focus on the important catalytic applications of noble metal particles confined in zeolites, and some other emerging applications in optics will also be included. For catalytic applications, we start to deal with the following two major issues: (i) the difference in the intrinsic electronic states and bonding capacities when going from single atoms to clusters and then to nanoparticles, and (ii) the dynamic characteristics of catalytic processes, which are directed by the metal–substrate interactions. In the latter case, the physicochemical properties can be significantly modified when bulk particles become smaller, especially in the form of subnanometric particles with less than 20 atoms or even single atoms on the solid supports.^108^, ^109^ These changes can be good or bad for a typical catalytic reaction. For the noble metal particles confined in zeolites, the intrinsic properties of metal particles will be naturally preserved and furthermore, zeolite frameworks act as shells providing more functional sites for cooperation with the confined metal sites.

### 4.1 Catalytic Applications of Zeolite‐Confined Noble Metal Particles

#### 4.1.1 Size‐Dependent Catalytic Properties

**Figure 7:**
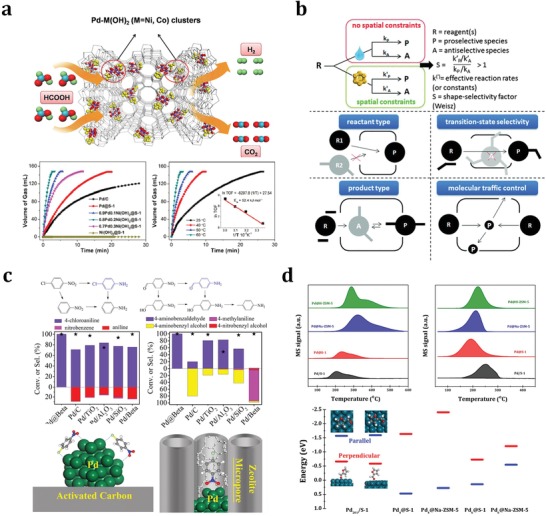
a) Schematic of formic acid decomposition catalyzed by Pd‐M(OH)_2_ confined in silicalite‐1 zeolite and the catalytic activity data. Adapted with permission.^112^ Copyright 2017, Elsevier Inc. b) The most basic definition of shape‐selectivity and three foremost types of shape‐selectivity along with molecular traffic control. Adapted with permission.^17^ Copyright 2016, The Royal Society of Chemistry. c) Substrate conversions and product selectivities for the hydrogenation of 4‐nitrochlorobenzene and 4‐nitrobenzaldehyde on Pd@MFI and reference Pd/C catalysts with the proposed models for 4‐nitrochlorobenzene adsorption. Adapted with permission.^60^ Copyright 2017, Wiley‐VCH. d) Temperature‐programmed desorption profiles of hydrogen (left) and furfur (right) on Pd/S‐1 and Pd@MFI with furfur adsorption configurations on Pd_201_ clusters in Pd/S‐1 and Pd_4_ or Pd_6_ clusters encapsulated in MFI zeolites. Adapted with permission.^46^ Copyright 2018, American Chemical Society.

Generally, the electronic properties of noble metal particles change significantly in the nanoscale range and, therefore, it can be expected that the metal nanoparticles will interact differently with reactants and show distinct reactivity with respect to bulk particles. For example, the size effects of supported noble metal nanoparticles on the catalytic oxidation of CO were illustrated.^110^ By a colloid chemical method, Pt nanoparticles with a narrow size distribution from 1 to 3 nm were deposited on FeOx support and employed as catalysts for CO oxidation, and the results revealed that Pt nanoparticles of around 2 nm exhibited the highest activity. Xiao and co‐workers investigated the total oxidation of volatile organic compounds using a series of size‐controllable Pt nanoparticles ranging from 1.3 to 2.3 nm on MFI zeolites as catalysts.^111^ It was concluded that Pt/MFI with size of 1.9 nm was most active in the reaction, due to a balance of Pt dispersion and Pt^0^ proportion. The size‐selected metal nanoparticles can be produced by confining them in designated spaces. Fortunately, zeolites with unique microporous structure are promising candidates in confining noble metal nanoparticles, which can be employed to study the size‐dependent effects in catalytic reactions. For example, Yu and co‐workers prepared ultrasmall Pd nanoparticles encapsulated in nanosized silicalite‐1, that is, Pd@MFI, for highly efficient H_2_ generation from the complete decomposition of formic acid under mild conditions.^50^ Notably, the Pd@MFI catalyst appeared to be most active for the reaction with turnover frequency values of 856 and 3027 h^−1^ at 298 and 323 K, respectively, much higher than reference catalysts Pd/C and Pd/silicalite‐1. This is a clear example of the size‐dependent effects, that is, reducing the size of Pd particles significantly to promote their catalytic activity. In the subsequent work, a series of subnanometric hybrid bimetallic clusters Pd‐M(OH)_2_ (M = Ni, Co) confined in silicalite‐1 were prepared and applied as catalysts for the decomposition of formic acid (**Figure** **7**a).^112^ Typically, the 0.8Pd0.2Ni(OH)_2_@silicalite‐1 catalyst afforded the highest initial turnover frequency value of up to 5803 h^−1^ toward the decomposition of formic acid without any additives at 333 K, which might be explained by both the size‐dependent effects and the electronic modifications. In fact, the size‐dependent catalytic properties of noble metal particles confined in zeolites can be observed for most metal@zeolites samples in various reactions.^40^, ^41^, ^44^, ^45^ However, these effects are often interfered by other factors, for example, electronic effects and spatial effects.^113^

#### 4.1.2 Sintering‐Resistance Properties

Catalyst sintering is a major cause of activity loss, and noble metal catalysts always suffer from serious sintering especially under harsh reaction conditions. Basically, the sintering of noble metal species can be described by the Ostwald ripening, the particle migration and coalescence models.^109^ To minimize sintering, it has been proposed to strengthen the metal–support interactions, and, more directly, coating the metals with protecting sheaths.^110^ Zeolite frameworks can be viewed as ideal protecting shells for noble metal particles. In fact, the most obvious advantage of noble metal particles confined in zeolites lies in their sintering‐resistance properties from the confinement effects of stable zeolite framework. Here, we will focus on the catalytic applications of metal@zeolites under the conditions likely to cause sintering. For example, Pt clusters confined in the internal cups and cages of MWW zeolites exhibited extremely high thermal stability even after calcination in air at up to 813 K and Pt@MWW showed much higher activity than Pt/MWW, which could be attributed to the confinement and stabilization of subnanometric Pt particles by MWW cavities.^54^ In addition, Pd nanoparticles confined in BEA zeolites, that is, Pd@BEA, were proved to be sintering‐resistant at 873–973 K under oxidizing atmosphere, and the uniform zeolite micropores also allowed the diffusion of reactant to contact with the confined Pd sites.^61^ As a result, the Pd@BEA samples exhibited very good long‐term stability, outperforming conventional Pd/BEA catalysts and commercial catalysts consisting of Pd nanoparticles on the surfaces of solid supports, in the catalytic conversion of C1 molecules, including the water–gas shift reaction, CO oxidation, methane reforming, and CO_2_ hydrogenation. Similarly, Pd nanoparticles were confined in MOR zeolite to improve their stability as methane oxidation catalysts against steam‐induced sintering at low temperatures of <773 K.^67^ Stable methane conversion can be maintained for 90 h in the presence of steam with the Pd@MOR catalyst.

#### 4.1.3 Substrate Shape‐Selective Catalysis

For zeolite‐encapsulated noble metal nanoparticles, the accessibility of the nanoparticles in the micropores and channels of zeolites is undoubtedly a problem and it significantly hinders the catalytic applications of nanoparticles when dealing with bulky reaction substrates. On the other hand, the limited diffusion of bulky reaction substrates can provide characteristic features for nanoparticles confined in zeolites as catalysts, which is now recognized as shape‐selectivity. The concept of shape‐selectivity (initially called as size‐selectivity) in zeolite catalysis was introduced in 1960 by scientists from Mobil,^114^ who observed the exceptional shape‐dependent catalytic performance of zeolites in the cracking of decane and the dehydration of butanols. Since then, this concept has shown tremendous impacts on the design of new catalytic processes in petrochemistry and refining. More simply, shape‐selectivity can be defined as a deviation, induced by constraints on molecular motion or product formation, in the product distribution relative to that obtained in absence of spatial constraints. A more generalized concept is the molecular traffic control of the reactants, products, and even reaction intermediates (Figure 7b).^17^, ^115^ Noble metal particles confined in zeolites, as typical core–shell materials, are feasible candidates for shape‐selective catalysis due to the shape‐selectivity from zeolite shells.^116^ Typically, the substrate shape‐selectivity of metal@zeolite can be adjusted by changing the diffusion of substrates within zeolite channels and the corresponding accessibility of confined noble metal particles. For example, Iglesia and co‐workers successfully encapsulated a series of noble metal clusters (Pt, Pd, Rh, Ir, and Ag) within LTA and GIS zeolites via direct hydrothermal synthesis,^40^ and the as‐prepared metal@zeolites showed high catalytic activity and superior shape‐selectivity in the oxidative dehydrogenation of alkanols (methanol, ethanol, and isobutanol) and the hydrogenation of alkenes (ethene and isobutene). Comparing the reaction rates in the hydrogenation of ethene and toluene and oxidative dehydrogenation of methanol and isobutanol catalyzed by metal@zeolites and unconstrained metal/SiO_2_, they disclosed that metal@zeolites could efficiently select appropriate reaction substrates according to their molecular sizes, that is, the sizes of reaction substrates should be smaller than the sizes of zeolite channels. Furthermore, Iglesia and co‐workers confirmed that the zeolite shells (GIS and ANA) could effectively protect the confined noble metal cores against poisoning by thiophene during ethene hydrogenation,^41^ which should be explained from the passing prohibition of the large organosulfur species through the small eight‐membered rings of zeolites. H_2_‐D_2_ isotopic exchange reaction in the presence and absence of H_2_S could give evidences on the effective protection of encapsulated clusters within sodalite cages against poisoning from toxic sulfur compounds. The H_2_‐D_2_ exchanging rates were much higher on Me@SOD than those on Me/SiO_2_ in the presence of H_2_S, because the H_2_S could not reach the surface of metal clusters confined in sodalite cages due to the limited diffusion through the channels of SOD zeolites. In the subsequent work, Iglesia and co‐workers reported the clear substrate shape‐selectivity of the encapsulated clusters within LTA and MFI zeolites in the oxidative dehydrogenation of alcohols,^45^ where the ethanol dehydrogenation rates were much higher than isobutanol originated from their different sizes. In another oxidation reaction, Egeblad and co‐workers found that the Au particles confined in mesoporous silicalite‐1, that is, Au@MFI, could catalyze the oxidation of benzaldehyde but could not catalyze the oxidation of 3,5‐di‐*tert*‐butylbenzaldehyde because the later one with much larger size could not diffuse through the channels of MFI zeolites to contact with the confined Au particles.^43^ Recently, Song and co‐workers reported the confinement of Pd particles in MFI zeolites and their applications as shape‐selective catalysts in the hydrogenation of carbonyl compounds.^49^ 3‐Methyl‐2‐butenal (0.38 × 0.62 nm) could be efficiently hydrogenated while diphenylacrylaldehyde (0.81 × 1.0 nm) could not, due to the precise size‐selection of reaction substrates by zeolite channels (0.53 × 0.56 nm). Moreover, Xiao and co‐workers designed a core–shell Pd@BEA catalyst to improve the performance of Pd nanoparticles in the hydrogenation of substituted nitroarenes into the corresponding anilines.^60^ The presence of BEA zeolite shell changed the adsorption of nitroarenes on Pd cores (Figure 7c), and therefore, showed significant impacts on the catalytic activity, selectivity, and catalyst lifetime.

To make a brief summary on this section, we expect the substrate shape‐selectivity to be a key advantage of noble metal catalysis confined in zeolites. The channels and pore openings of zeolites can reasonably select the reaction substrates, according to their sizes and shapes, for contacting with confined noble metal particles. In such a way, desired substrate shape‐selectivity can be achieved with reactions catalyzed by metal@zeolites. On the other hand, the block of bulky poisoning reagents by the channels and pore openings of zeolites can effectively prevent the deactivation of confined noble metal particles due to poisoning. Besides, the properties of substrate shape‐selective catalysis can be seen as an overwhelming evidence on the successful confinement of noble metal particles in zeolites.

#### 4.1.4 Catalysis Modulation by Zeolite Microenvironment

In addition to the confined noble metal particles, the aluminosilicate zeolites are not inert supports and they can provide fine‐tuned acid–base sites, special electronic interactions, and well‐defined channels for catalytic processes.^117^, ^118^ This is very complicated due to the interference of multiple factors. However, it should not be ignored due to its significant impacts on the catalytic properties of confined noble metal particles. For example, the acid–base sites in zeolites are in close proximity with the encapsulated nanoparticles, and can further alter their properties or even build cooperative catalysts together. Here, all these factors are simply categorized in zeolite microenvironment, which can significantly modulate the catalytic activity and, more importantly, selectivity of confined noble metal particles in certain reactions. Li and co‐workers reported the in situ encapsulation of Pd nanoparticles in MFI zeolites and the construction of Pd@MFI catalysts on the basis of encapsulated Pd nanoparticles and MFI zeolite microenvironment.^46^ In the hydroconversion of furfural, different products, for example, furan, furfural alcohol, and 1,5‐pentanediol, were obtained when silicalite‐1, Na‐ZSM‐5, and H‐ZSM‐5 were employed as hosts of palladium nanoparticles, respectively. Density functional theory calculations and spectroscopy investigations clearly revealed that both the adsorption of furfural and the activation of hydrogen were significantly affected by zeolite microenvironment (Figure 7d), and the selectivity modulation of Pd catalysis by tuning the zeolite microenvironment was thus established. Xiao and co‐workers also reported the use of core–shell structural Pd@S‐1, that is, Pd encapsulated in silicalite‐1 zeolite, as a catalyst for furfural hydroconversion.^47^ Typically, the furan selectivity over the Pd@S‐1 was as high as 98.7% at furfural conversion of 91.3%, in great contrast to the furan selectivity of 5.6% achieved with Pd/S‐1. This was ascribed to the distinguishable mass transfer of the hydrogenated products in the zeolite micropores. In the further work, they further changed the wettability of the zeolite environment by functionalizing the silanol groups in the zeolite framework.^119^ The as‐prepared sample, labeled as Pd@S‐1‐OH, exhibited extraordinary selectivity toward the formation of furan in furfural hydroconversion, giving furan selectivity at 99.9% at complete conversion of furfural due to specific zeolite wettability.

As discussed above, changing the zeolite microenvironment of confining noble metal particles represents a simple but efficient strategy to the selectivity modulation in heterogeneous catalysis. However, the zeolite microenvironment itself is complicated and hard to clarify, not to mention about the more complicated metal@zeolites. In this context, special attention should be paid in discussing the impacts of zeolite microenvironment on the confining noble metal particles.

### 4.2 Optical Properties of Zeolite‐Confined Noble Metal Particles

The noble metal clusters can show molecule (atom)‐like behaviors in terms of their electronic transitions. Therefore, in addition to their catalytic applications, noble metal clusters confined in zeolites are playing an increasingly important role in other processes, for example, optics applications. The unique optical properties of small metal clusters contribute to an intriguing research topic for physical scientists. In contrast to bulk metals, small metal clusters (M*_n_^m^* ^+^ with *n* < 100 and *m* < *n*) are known to have discrete electronic energy states, and accordingly exhibit molecule‐like behaviors.^120^ For specific clusters of noble metals, the cluster sizes and charges can show significant impacts on the emission colors and the bright emissions are usually excited within the UV–visible range.^121^ The synthesis of monodisperse noble metal clusters without tendency toward aggregation represents a key challenge for optical applications.^122^ Generally, the selective production of specific metal clusters can be achieved by varying the metal–guest and structure–host properties. Undoubtedly, zeolites with versatile framework structures and cages become attractive hosts for noble metal clusters. Ag clusters confined in zeolites, that is, Ag*_n_*@zeolites, have been extensively investigated considering the ease of preparation of Ag*_n_*@zeolites (see Section 2.4) as well as their well‐defined crystal structures. As for the zeolite hosts, most studies focus on LTA and FAU topologies, both with sodalite cages but different secondary building units interconnecting these cages (double four‐rings for LTA and double six‐rings for FAU). In the very early days, Gellens et al. investigated the nature of the charged Ag clusters in zeolites A (LTA), X and Y (FAU) and their dehydrated forms.^62^ All samples initially looked gray and became yellow upon dehydration and oxygen treatment at 693 K. The higher the Al content, the brighter the color that was observed. They concluded that, in both structure types, linear charged clusters of limited sizes were formed upon dehydration and oxidation, which were responsible for the yellow color formation. They also investigated the electronic structure of the Ag clusters confined in zeolites by molecular orbital calculations and revealed that Ag_3_ was a molecule with a strong silver–silver bond and a linear or nearly linear geometry while Ag_6_ was 3D with the low‐energy bands shifted to lower energy.^24^ The interaction between the two Ag_3_ units was strong, and differently, the formation of the Ag_6_ cluster was determined by metal–metal interactions rather than metal–lattice interactions.

**Figure 8:**
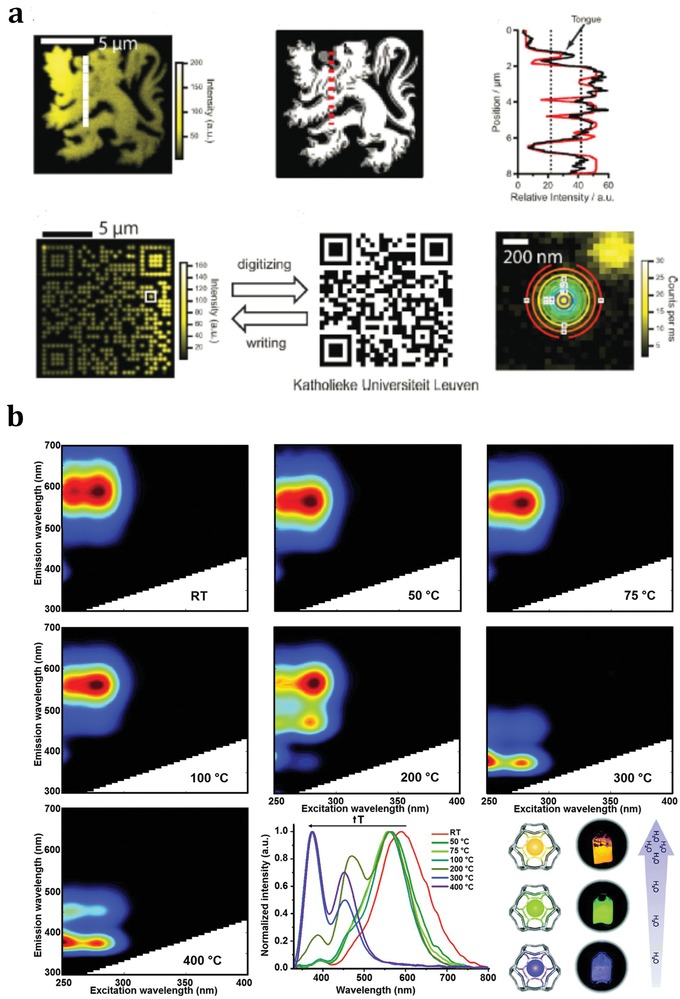
a) Optical encoding of Ag@zeolite microcarriers via two‐photon activation. Adapted with permission.^124^ Copyright 2010, Wiley‐VCH. b) Excitation‐emission profiles of Ag@Li‐LTA after heat‐treatment at different temperatures with a compilation of the normalized emission profiles obtained upon excitation at 260 nm and schematic of the color change of real samples with respect to the water content. Adapted with permission.^71^ Copyright 2015, The Royal Society of Chemistry.

It can be seen that Ag_n_@zeolites are promising candidates for optical applications. The structures, sizes, and charges of the confined Ag clusters were sensitive to the conditions of synthesis, the structures of zeolite hosts, and the activation conditions. The activation for the Ag clusters confined in zeolite A, a type of d^10^‐zeolite materials, at room temperature under high vacuum was already sufficient to produce the yellow form of Ag^+^ *x*Na^+^_12−_ *_x_*A.^123^ The fully reversible color change, caused by the hydration of Ag*_n_*@LTA, was attributed to the electronic charge transfer transitions from the lone pairs of zeolite framework oxygen atoms to the empty 5s orbitals of Ag^+^ ions, denoted as O(n)→Ag^+^(5s), a kind of π back bonding. The white Ag*_n_*@LTA turned yellow upon activation in high vacuum at room temperature, and further turned brick red in high vacuum at 473 K, which could be well explained by UV–visible spectroscopy. Typically, six‐ and eight‐ring coordinated Ag^+^ gave rise to electronic transitions in the near‐UV region, while the four‐ring coordinated Ag^+^ showed an absorption in the visible region, namely, at 450 nm with the apparent color of deep yellow. Another interesting point was that only samples with four‐ring coordinated Ag^+^ could contribute to the 520 nm band with the apparent color of red, if a second Ag^+^ was not too far away at a six‐ring site so that they could interact to develop a corresponding low lying state. Sels and co‐workers^69^, ^71^ described a convenient route for the creation of stable oligoatomic Ag clusters within LTA zeolite matrix via a simple ion‐exchange process followed by thermal or photochemical reduction. These clusters showed interesting emissive properties that might find widespread applications, for instance, as wavelength converters in fluorescence lamps or solar cells, or as bright and photostable biocompatible labels. Ag@LTA was further applied in optical encoding (**Figure** **8**a),^124^ where the encoded patterns were stable over extended periods of time, thanks to the confined location of fluorescent Ag clusters and the protective environment of zeolite matrix.

Ag@zeolite materials also exhibit other optical characteristics such as high external quantum efficiencies and large Stokes shifts.^24^, ^62^ The selective formation of luminescent Ag clusters within zeolite matrix could be achieved by tuning Ag guest and zeolite host interaction. For example, Ag@Li‐LTA displayed better luminescence performance than Ag@Na‐LTA samples, and water‐responsive photoluminescence properties were observed for the first time. Yellow, green, and blue emissions were observed in Ag@Li‐LTA materials with different hydration levels.^87^ As shown in Figure 8b,^71^ a broad emission band centered at 590 nm (yellow emission) was observed at room temperature (about 200 water molecules per normalized unit cell) when the sample was excited at 260 and 280 nm. This emission band was slightly blueshifted by about 30 nm (green emission) when the sample was heated to 323 K (about 180 water molecules per normalized unit cell). The green emission could be observed in the samples heated to 348 and 373 K (about 120 and 50 water molecules per normalized unit cell, respectively) with reducing emission intensity excited at 260 nm. Hopefully, the Ag@Li‐LTA materials might be used to build luminescence‐based humidity sensors.

For a direct view, a brief summary on the confined noble metal particles, zeolite framework, and applications of metal@zeolites is shown in **Table** **2**.

**Table 2:** Metal@zeolite samples and their typical applications

Entry	Metal	Size [nm]	Zeolite	Applications	Ref.
1	Pt, Ru, Rh	1.3–1.7	MFI	Catalytic hydrogenation of arenes	32
2	Pt	≈1	CHA	Catalytic hydrogenation of olefin	33
3	Pt, Pd, Ru, Rh	1.3–1.8	SOD, GIS, ANA	Catalytic dehydrogenation of alkanols	41
4	Pt	1.3–1.8	GIS, ANA	Catalytic hydrogenation of ethene and toluene	41
5	Au	1–2	MFI	Catalytic oxidation of aldehydes	43
6	Pt, Pd, Ir, Rh, Ag	1.2–1.9	LTA	Catalytic dehydrogenation of alcohols	45
7	Pd	≈1.7	MFI	Catalytic hydroconversion of furfural	46
8	Pd	3.7–11.7	MFI	Catalytic hydrogenation of furfural	47
9	Pd	≈1.1	MFI	Catalytic decomposition of formic acid	50
10	Pt	0.4–0.7	MWW	Catalytic hydrogenation of propene	54
11	Pd	≈1.7	BEA	Catalytic hydrogenation of nitroarene	58
12	Ag	≈0.3	LTA	Luminescence‐based humidity sensors	71
13	Ag	≈0.3	LTA	Photoluminescence emission	87
14	Ag	≈0.3	LTA	Optical encoding	24

## 5 Concluding Remarks and Perspectives

Noble metal nanoparticles or subnanometric particles confined in zeolites represent an important type of functional materials with typical core–shell structure, that is, noble metal particles as cores and zeolites as shells. This type of materials have been known for decades and recently become a research hotspot due to their emerging applications in various fields. Remarkable achievements have been made dealing with the synthesis, characterization, and applications of noble metal particles confined in zeolites, that is, metal@zeolites. In this article, the most representative research progresses on metal@zeolites are briefly reviewed, aiming to boost further researches on this topic. For the synthesis of metal@zeolites, various strategies, such as direct synthesis from inorganic or ligand‐assisted noble metal precursors, multistep postsynthesis encapsulation, and ion‐exchange followed by reduction, are introduced and compared. For the characterization of metal@zeolites, several most useful techniques, such as electron microscopy, X‐ray based spectroscopy, infrared and fluorescence emission spectroscopy, have been recommended to check the successful confinement of noble metal particles in zeolite matrix and their unique physiochemical properties. For the applications of metal@zeolites, catalysis and optics have been involved with emphasis on catalytic applications including the size‐dependent catalytic properties, the sintering‐resistance properties, the substrate shape‐selective catalysis, and catalysis modulation by zeolite microenvironment.

**Figure 9:**
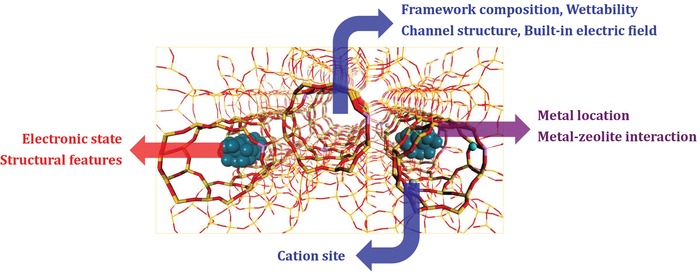
Major factors relevant to the properties of noble metal particles confined in zeolites.

Despite current achievements, many issues on the synthesis, characterization, and applications of metal@zeolites are still to be solved. Nevertheless, metal@zeolites have already demonstrated their great potential for practical applications, which are now attracting more and more attention. It is well known that the performance of materials is determined by their properties. As for metal@zeolites, the properties or so‐called functionalities may come from the confined noble metal particles, the zeolites hosts, and their interactions. The major factors that can produce or influence properties are summarized in **Figure** **9**, from which the complexity of metal@zeolites is clearly seen. Actually, the properties of metal@zeolite materials come from the balance of various factors, which are difficult to interpret. In this context, we should focus on the decisive factors or, more safely, treat the metal@zeolites as a whole rather than confined metal plus zeolite hosts. According to the literature and our own experience, some specific challenges and opportunities on noble metal particles confined in zeolites are briefly prospected as follows. (1) General synthesis strategies to metal@zeolites. Since current synthesis strategies are more or less limited by both the types of confined noble metals and zeolites hosts, general strategies to confining noble metal particles in zeolites are urgently desired, which are the basis for subsequent researches. Low‐cost synthesis route with good reproducibility is mostly desired for industrial production. (2) Fine structure and exact location of particles in zeolites. While the structure of isolated particles can be clearly identified by various techniques, it is challenging to characterize the fine structure of confined noble metal particles due to the unavoidable interference from zeolite framework. Besides, noble metal particles can be confined in the channels and cages of zeolite or even attached to zeolite framework, which are difficult to be distinguished. To solve these problems, advanced characterization technique should be applied and, for example, high‐resolution electron microscopy might be very useful in providing direct evidence on both the fine structure and exact location of confined particles. (3) Formation of confined clusters. Thanks to the persistent efforts in the synthesis of metal@zeolites, small noble metal nanoparticles or subnanometric particles with uniform size of <2 nm have been successfully confined in zeolites. To make a step forward, the formation of zeolite‐confined noble metal clusters is expected. Currently, the formation of Ag clusters with well‐defined structure in LTA and FAU zeolites has been accomplished, which should be extended to other noble metals and other zeolite hosts. (4) Irreplaceable applications of metal@zeolites. Metal@zeolites have found widespread applications in catalysis and optics, and exhibited significant advantages over other types of materials. In catalysis, the activity, selectivity, and stability in certain reactions can be significantly improved by using metal@zeolites as catalysts. Nevertheless, more irreplaceable catalytic applications are expected for this type of materials. To achieve this goal, the more efficient utilizations of zeolite hosts, that is, channel systems and functional sites, and metal–zeolite interactions are proposed.

## Conflict of Interest

The authors declare no conflict of interest.

## References

[1] J. Lu , B. Fu , M. C. Kung , G. Xiao , J. W. Elam , H. H. Kung , P. C. Stair , Science 2012, 335, 1205. 22403386 10.1126/science.1212906

[2] J. M. Campelo , D. Luna , R. Luque , J. M. Marinas , A. A. Romero , ChemSusChem 2009, 2, 18. 19142903 10.1002/cssc.200800227

[3] A. Kulkarni , R. J. Lobo‐Lapidus , B. C. Gates , Chem. Commun. 2010, 46, 5997. 10.1039/c002707n 20661500

[4] R. G. Rao , R. Blume , T. W. Hansen , E. Fuentes , K. Dreyer , S. Moldovan , O. Ersen , D. D. Hibbitts , Y. J. Chabal , R. Schlögl , J.‐P. Tessonnier , Nat. Commun. 2017, 8, 340. 28835704 10.1038/s41467-017-00421-x PMC5569089

[5] G. Prieto , J. Zečević , H. Friedrich , K. P. De Jong , P. E. De Jongh , Nat. Mater. 2013, 12, 34. 23142841 10.1038/nmat3471

[6] P. Munnik , P. E. De Jongh , K. P. De Jong , Chem. Rev. 2015, 115, 6687. 26088402 10.1021/cr500486u

[7] A. Corma , H. Garcia , Chem. Soc. Rev. 2008, 37, 2096. 18762848 10.1039/b707314n

[8] G. A. Somorjai , J. Y. Park , Chem. Soc. Rev. 2008, 37, 2155. 18818818 10.1039/b719148k

[9] M. Crespo‐Quesada , A. Yarulin , M. Jin , Y. Xia , L. Kiwi‐Minsker , J. Am. Chem. Soc. 2011, 133, 12787. 21749155 10.1021/ja204557m

[10] T. W. Hansen , A. T. Delariva , S. R. Challa , A. K. Datye , Acc. Chem. Res. 2013, 46, 1720. 23634641 10.1021/ar3002427

[11] S. R. Challa , A. T. Delariva , T. W. Hansen , S. Helveg , J. Sehested , P. L. Hansen , F. Garzon , A. K. Datye , J. Am. Chem. Soc. 2011, 133, 20672. 22087502 10.1021/ja208324n

[12] T. Harada , S. Ikeda , Y. H. Ng , T. Sakata , H. Mori , T. Torimoto , M. Matsumura , Adv. Funct. Mater. 2008, 18, 2190.

[13] D. Farrusseng , A. Tuel , New J. Chem. 2016, 40, 3933.

[14] G. Riahi , D. Guillemot , M. Polisset‐Thfoin , A. A. Khodadadi , J. Fraissard , Catal. Today 2002, 72, 115.

[15] J.‐C. Kim , S. Lee , K. Cho , K. Na , C. Lee , R. Ryoo , ACS Catal. 2014, 4, 3919.

[16] D. Xu , H. Lv , B. Liu , Front. Chem. 2018, 6, 550. 30474024 10.3389/fchem.2018.00550 PMC6238153

[17] T. Ennaert , J. Van Aelst , J. Dijkmans , R. De Clercq , W. Schutyser , M. Dusselier , D. Verboekend , B. F. Sels , Chem. Soc. Rev. 2016, 45, 584. 26691750 10.1039/c5cs00859j

[18] B. M. Weckhuysen , J. Yu , Chem. Soc. Rev. 2015, 44, 7022. 26401967 10.1039/c5cs90100f

[19] X. Meng , F. S. Xiao , Chem. Rev. 2014, 114, 1521. 24187944 10.1021/cr4001513

[20] V. Van Speybroeck , K. Hemelsoet , L. Joos , M. Waroquier , R. G. Bell , C. R. A. Catlow , Chem. Soc. Rev. 2015, 44, 7044. 25976164 10.1039/c5cs00029g

[21] D. Fodor , T. Ishikawa , F. Krumeich , J. A. Van Bokhoven , Adv. Mater. 2015, 27, 1919. 25645289 10.1002/adma.201404628

[22] J. Zhang , L. Wang , L. Zhu , Q. Wu , C. Chen , X. Wang , Y. Ji , X. Meng , F. S. Xiao , ChemSusChem 2015, 8, 2867. 26043428 10.1002/cssc.201500261

[23] G. C. Chinchen , P. J. Denny , J. R. Jennings , M. S. Spencer , K. C. Waugh , Appl. Catal. 1988, 36, 1.

[24] L. R. Gellens , W. J. Mortier , R. Lissillour , A. Le Beuze , J. Phys. Chem. 1982, 86, 2509.

[25] L. Liu , A. Corma , Chem. Rev. 2018, 118, 4981. 29658707 10.1021/acs.chemrev.7b00776 PMC6061779

[26] N. Kosinov , C. Liu , E. J. M. Hensen , E. A. Pidko , Chem. Mater. 2018, 30, 3177. 29861546 10.1021/acs.chemmater.8b01311 PMC5973782

[27] N. Wang , Q. Sun , J. Yu , Adv. Mater. 2019, 31, 1803966. 10.1002/adma.201803966 30276888

[28] H. Zhang , M. Jin , Y. Xia , Angew. Chem., Int. Ed. 2012, 51, 7656. 10.1002/anie.201201557 22639064

[29] H. Cheng , N. Yang , Q. Lu , Z. Zhang , H. Zhang , Adv. Mater. 2018, 30, 1707189. 10.1002/adma.201707189 29658155

[30] M. Moliner , F. Rey , A. Corma , Angew. Chem., Int. Ed. 2013, 52, 13880. 10.1002/anie.201304713 24115577

[31] A. Corma , M. E. Davis , ChemPhysChem 2004, 5, 304.

[32] S. Goel , S. I. Zones , E. Iglesia , J. Am. Chem. Soc. 2014, 136, 15280. 25314634 10.1021/ja507956m

[33] M. Moliner , J. E. Gabay , C. E. Kliewer , R. T. Carr , J. Guzman , G. L. Casty , P. Serna , A. Corma , J. Am. Chem. Soc. 2016, 138, 15743. 27934002 10.1021/jacs.6b10169

[34] C. S. Cundy , P. A. Cox , Chem. Rev. 2003, 103, 663. 12630849 10.1021/cr020060i

[35] M. Moliner , ISRN Mater. Sci. 2012, 2012, 1.

[36] B. Zhan , M. A. White , T. Sham , J. A. Pincock , R. J. Doucet , K. V. R. Rao , K. N. Robertson , T. S. Cameron , J. Am. Chem. Soc. 2003, 125, 2195. 12590547 10.1021/ja0282691

[37] S. Altwasser , R. Gläser , A. S. Lo , P. H. Liu , K. J. Chao , J. Weitkamp , Microporous Mesoporous Mater. 2006, 89, 109.

[38] B. Z. Zhan , E. Iglesia , Angew. Chem., Int. Ed. 2007, 46, 3697. 10.1002/anie.200700128 17410631

[39] J. Guzman , B. C. Gates , Dalton Trans. 2003, 3303.

[40] Z. Wu , S. Goel , M. Choi , E. Iglesia , J. Catal. 2014, 311, 458.

[41] S. Goel , Z. Wu , S. I. Zones , E. Iglesia , J. Am. Chem. Soc. 2012, 134, 17688. 23016946 10.1021/ja307370z

[42] K. J. Balkus , A. G. Gabrielov , J. Inclusion Phenom. Mol. Recognit. Chem. 1995, 21, 159.

[43] A. B. Laursen , K. T. Højholt , L. F. Lundegaard , S. B. Simonsen , S. Helveg , F. Schüth , M. Paul , J. D. Grunwaldt , S. Kegnœs , C. H. Christensen , K. Egeblad , Angew. Chem., Int. Ed. 2010, 49, 3504. 10.1002/anie.200906977 20391442

[44] T. Otto , S. I. Zones , E. Iglesia , J. Catal. 2016, 339, 195.

[45] M. Choi , Z. Wu , E. Iglesia , J. Am. Chem. Soc. 2010, 132, 9129. 20536236 10.1021/ja102778e

[46] Y. Chai , S. Liu , Z. J. Zhao , J. Gong , W. Dai , G. Wu , N. Guan , L. Li , ACS Catal. 2018, 8, 8578.

[47] C. Wang , L. Wang , J. Zhang , H. Wang , J. P. Lewis , F. S. Xiao , J. Am. Chem. Soc. 2016, 138, 7880. 27308846 10.1021/jacs.6b04951

[48] T. L. Cui , W. Y. Ke , W. B. Zhang , H. H. Wang , X. H. Li , J. S. Chen , Angew. Chem., Int. Ed. 2016, 55, 9178. 10.1002/anie.201602429 27346582

[49] C. Liu , J. Liu , S. Yang , C. Cao , W. Song , ChemCatChem 2016, 8, 1279.

[50] N. Wang , Q. Sun , R. Bai , X. Li , G. Guo , J. Yu , J. Am. Chem. Soc. 2016, 138, 7484. 27248462 10.1021/jacs.6b03518

[51] L. Ren , L. Zhu , C. Yang , Y. Chen , Q. Sun , H. Zhang , C. Li , F. Nawaz , X. Meng , F. S. Xiao , Chem. Commun. 2011, 47, 9789. 10.1039/c1cc12469b 21625721

[52] S. I. Zones , J. Chem. Soc., Faraday Trans. 1991, 87, 3709.

[53] J. Gu , Z. Zhang , P. Hu , L. Ding , N. Xue , L. Peng , X. Guo , M. Lin , W. Ding , ACS Catal. 2015, 5, 6893.

[54] L. Liu , U. Díaz , R. Arenal , G. Agostini , P. Concepción , A. Corma , Nat. Mater. 2017, 16, 132. 27669051 10.1038/nmat4757

[55] L. Liu , D. N. Zakharov , R. Arenal , P. Concepcion , E. A. Stach , A. Corma , Nat. Commun. 2018, 9, 574. 29422522 10.1038/s41467-018-03012-6 PMC5805776

[56] S. Li , T. Boucheron , A. Tuel , D. Farrusseng , F. Meunier , Chem. Commun. 2014, 50, 1824. 10.1039/c3cc48648f 24398573

[57] S. Li , L. Burel , C. Aquino , A. Tuel , F. Morfin , J. L. Rousset , D. Farrusseng , Chem. Commun. 2013, 49, 8507. 10.1039/c3cc44843f 23942629

[58] S. Li , A. Tuel , J. L. Rousset , F. Morfin , M. Aouine , L. Burel , F. Meunier , D. Farrusseng , ChemNanoMat 2016, 2, 534.

[59] Y. Kamimura , S. Tanahashi , K. Itabashi , A. Sugawara , T. Wakihara , A. Shimojima , T. Okubo , J. Phys. Chem. C 2011, 115, 744.

[60] J. Zhang , L. Wang , Y. Shao , Y. Wang , B. C. Gates , F. S. Xiao , Angew. Chem., Int. Ed. 2017, 56, 9747. 10.1002/anie.201703938 28503914

[61] J. Zhang , L. Wang , B. Zhang , H. Zhao , U. Kolb , Y. Zhu , L. Liu , Y. Han , G. Wang , C. Wang , D. S. Su , B. C. Gates , F. S. Xiao , Nat. Catal. 2018, 1, 540.

[62] L. R. Gellens , W. J. Mortier , J. B. Uytterhoeven , Zeolites 1981, 1, 11.

[63] J. D. Graaf , A. J. V. Dillen , K. P. D. Jong , D. C. Koningsberger , J. Catal. 2001, 203, 307.

[64] J. Cai , H. Ma , J. Zhang , Q. Song , Z. Du , Y. Huang , J. Xu , Chem. ‐ Eur. J. 2013, 19, 14215. 23999985 10.1002/chem.201301735

[65] W. J. Roth , P. Nachtigall , R. E. Morris , J. Čejka , Chem. Rev. 2014, 114, 4807. 24555638 10.1021/cr400600f

[66] Z. Zhao , Y. Li , M. Feyen , R. McGuire , U. Müller , W. Zhang , ChemCatChem 2018, 10, 2254.

[67] A. W. Petrov , D. Ferri , F. Krumeich , M. Nachtegaal , J. A. V. Bokhoven , O. Kröcher , Nat. Commun. 2018, 9, 2545. 29959324 10.1038/s41467-018-04748-x PMC6026177

[68] T. Iida , D. Zanchet , K. Ohara , T. Wakihara , Y. Román‐Leshkov , Angew. Chem., Int. Ed. 2018, 57, 6454. 10.1002/anie.201800557 29575492

[69] G. De Cremer , E. Coutiño‐Gonzalez , M. B. J. Roeffaers , B. Moens , J. Ollevier , M. Van Der Auweraer , R. Schoonheydt , P. A. Jacobs , F. C. De Schryver , J. Hofkens , D. E. De Vos , B. F. Sels , T. Vosch , J. Am. Chem. Soc. 2009, 131, 3049. 19209854 10.1021/ja810071s

[70] A. M. Fonseca , I. C. Neves , Microporous Mesoporous Mater. 2013, 181, 83.

[71] E. Coutino‐Gonzalez , W. Baekelant , D. Grandjean , M. B. J. Roeffaers , E. Fron , M. S. Aghakhani , N. Bovet , M. V. D. Auweraer , P. Lievens , T. Vosch , B. sels , J. Hofkens , J. Mater. Chem. C 2015, 3, 11857.

[72] G. D. Cremer , E. Coutiño‐Gonzalez , M. B. J. Roeffaers , D. E. D. Vos , J. Hofkens , T. Vosch , B. F. Sels , ChemPhysChem 2010, 11, 1627. 20349496 10.1002/cphc.201000065

[73] O. Fenwick , E. Coutiño‐Gonzalez , D. Grandjean , W. Baekelant , F. Richard , S. Bonacchi , D. De Vos , P. Lievens , M. Roeffaers , J. Hofkens , P. Samorì , Nat. Mater. 2016, 15, 1017. 27270964 10.1038/nmat4652

[74] D. Grandjean , E. Coutiño‐Gonzalez , N. T. Cuong , E. Fron , W. Baekelant , S. Aghakhani , P. Schlexer , F. D'acapito , D. Banerjee , M. B. J. Roeffaers , M. T. Nguyen , J. Hofkens , P. Lievens , Science 2018, 361, 686. 30115807 10.1126/science.aaq1308

[75] E. Coutino‐Gonzalez , M. B. J. Roeffaers , B. Dieu , G. De Cremer , S. Leyre , P. Hanselaer , W. Fyen , B. Sels , J. Hofkens , J. Phys. Chem. C 2013, 117, 6998.

[76] J. K. Nørskov , T. Bligaard , J. Rossmeisl , C. H. Christensen , Nat. Chem. 2009, 1, 37. 21378799 10.1038/nchem.121

[77] H. Friedrich , P. E. De Jongh , A. J. Verkleij , K. P. De Jong , Chem. Rev. 2009, 109, 1613. 19301813 10.1021/cr800434t

[78] C. Copéret , W. C. Liao , C. P. Gordon , T. C. Ong , J. Am. Chem. Soc. 2017, 139, 10588. 28657741 10.1021/jacs.6b12981

[79] J. C. Groen , L. A. A. Peffer , J. Pérez‐Ramírez , Microporous Mesoporous Mater. 2003, 60, 1.

[80] M. K. Miller , R. G. Forbes , Mater. Charact. 2009, 60, 461.

[81] F. C. Jentoft , Ultraviolet‐Visible‐Near Infrared Spectroscopy in Catalysis, Elsevier Inc., Amsterdam 2009.

[82] J. A. van Bokhoven , C. Lamberti , Coord. Chem. Rev. 2014, 277, 275.

[83] C. Martinez‐Macias , P. Xu , S. J. Hwang , J. Lu , C. Y. Chen , N. D. Browning , B. C. Gates , ACS Catal. 2014, 4, 2662.

[84] A. Villa , N. Dimitratos , C. E. Chan‐Thaw , C. Hammond , G. M. Veith , D. Wang , M. Manzoli , L. Prati , G. J. Hutchings , Chem. Soc. Rev. 2016, 45, 4953. 27200435 10.1039/c5cs00350d

[85] W. Gao , Z. D. Hood , M. Chi , Acc. Chem. Res. 2017, 50, 787. 28207240 10.1021/acs.accounts.6b00596

[86] M. Rozmus , M. Blicharski , S. Dymek , J. Microsc. 2006, 224, 58. 17100907 10.1111/j.1365-2818.2006.01663.x

[87] A. Mayoral , T. Carey , P. A. Anderson , A. Lubk , I. Diaz , Angew. Chem., Int. Ed. 2011, 50, 11230. 10.1002/anie.201105450 21956896

[88] D. Yang , P. Xu , N. D. Browning , B. C. Gates , J. Phys. Chem. Lett. 2016, 7, 2537. 27315020 10.1021/acs.jpclett.6b01153

[89] E. Bayram , J. Lu , C. Aydin , N. D. Browning , S. Özkar , E. Finney , B. C. Gates , R. G. Finke , ACS Catal. 2015, 5, 3514.

[90] J. D. Kistler , N. Chotigkrai , P. Xu , B. Enderle , P. Praserthdam , C. Y. Chen , N. D. Browning , B. C. Gates , Angew. Chem., Int. Ed. 2014, 53, 8904. 10.1002/anie.201403353 24986134

[91] B. C. Gates , M. Flytzani‐Stephanopoulos , D. A. DIxon , A. Katz , Catal. Sci. Technol. 2017, 7, 4259.

[92] A. Anson , Y. Maham , C. C. H. Lin , T. M. Kuznicki , S. M. Kuznicki , J. Nanosci. Nanotechnol. 2009, 9, 3134. 19452980 10.1166/jnn.2009.044

[93] J. Singh , C. Lamberti , J. A. Van Bokhoven , Chem. Soc. Rev. 2010, 39, 4754. 20981379 10.1039/c0cs00054j

[94] M. Newville , Rev. Mineral. Geochem. 2014, 78, 33.

[95] J. J. Rehr , J. J. Kas , F. D. Vila , M. P. Prange , K. Jorissen , Phys. Chem. Chem. Phys. 2010, 12, 5503. 20445945 10.1039/b926434e

[96] L. X. Chen , X. Zhang , M. L. Shelby , Chem. Sci. 2014, 5, 4136.

[97] S. Bordiga , E. Groppo , G. Agostini , J. A. van Bokhoven , C. Lamberti , Chem. Rev. 2013, 113, 1736. 23444971 10.1021/cr2000898

[98] T. Yamamoto , S. Takenaka , T. Tanaka , T. Baba , J. Phys.: Conf. Ser. 2009, 190, 012171.

[99] R. C. Nelson , J. T. Miller , Catal. Sci. Technol. 2012, 2, 461.

[100] J. C. Fierro‐Gonzalez , Y. Hao , B. C. Gates , J. Phys. Chem. 2007, 111, 6645.

[101] A. I. Frenkel , C. W. Hills , R. G. Nuzzo , J. Phys. Chem. B 2001, 105, 12689.

[102] P. Glatzel , U. Bergmann , Coord. Chem. Rev. 2005, 249, 65.

[103] P. Glatzel , M. Sikora , G. Smolentsev , M. Fernández‐García , Catal. Today 2009, 145, 294.

[104] B. Tang , W. Dai , G. Wu , N. Guan , L. Li , M. Hunger , ACS Catal. 2014, 4, 2801.

[105] Y. Chai , L. Xie , Z. Yu , W. Dai , G. Wu , N. Guan , L. Li , Microporous Mesoporous Mater. 2018, 264, 230.

[106] S. M. Rogers , C. R. A. Catlow , C. E. Chan‐Thaw , A. Chutia , N. Jian , R. E. Palmer , M. Perdjon , A. Thetford , N. Dimitratos , A. Villa , P. P. Wells , ACS Catal. 2017, 7, 2266.

[107] M. Lepage , T. Visser , F. Soulimani , A. M. Beale , A. Iglesias‐Juez , A. M. J. Van Der Eerden , B. M. Weckhuysen , J. Phys. Chem. C 2008, 112, 9394.

[108] A. Corma , P. Concepción , M. Boronat , M. J. Sabater , J. Navas , M. J. Yacaman , E. Larios , A. Posadas , M. A. López‐Quintela , D. Buceta , E. Mendoza , G. Guilera , A. Mayoral , Nat. Chem. 2013, 5, 775. 23965680 10.1038/nchem.1721

[109] S. B. Simonsen , I. Chorkendorff , S. Dahl , M. Skoglundh , J. Am. Chem. Soc. 2010, 132, 7968. 20481529 10.1021/ja910094r

[110] N. An , S. Li , P. N. Duchesne , P. Wu , W. Zhang , J. F. Lee , S. Cheng , P. Zhang , M. Jia , W. Zhang , J. Phys. Chem. C 2013, 117, 21254.

[111] C. Chen , F. Chen , L. Zhang , S. Pan , C. Bian , X. Zheng , X. Meng , F. S. Xiao , Chem. Commun. 2015, 51, 5936. 10.1039/c4cc09383f 25738186

[112] Q. Sun , N. Wang , Q. Bing , R. Si , J. Liu , R. Bai , P. Zhang , M. Jia , J. Yu , Chem 2017, 3, 477.

[113] S. H. Joo , J. Y. Park , C. K. Tsung , Y. Yamada , P. Yang , G. A. Somorjai , Nat. Mater. 2009, 8, 126. 19029893 10.1038/nmat2329

[114] V. J. Frilette , P. B. Weisz , R. L. Golden , J. Catal. 1962, 1, 301.

[115] T. F. Degnan , J. Catal. 2003, 216, 32.

[116] B. Smit , T. L. M. Maesen , Nature 2008, 451, 671. 18256663 10.1038/nature06552

[117] C. S. Cundy , P. A. Cox , Microporous Mesoporous Mater. 2005, 82, 1.

[118] C. Paolucci , I. Khurana , A. A. Parekh , S. Li , A. J. Shih , H. Li , J. R. Di Iorio , J. D. Albarracin‐Caballero , A. Yezerets , J. T. Miller , W. N. Delgass , F. H. Ribeiro , W. F. Schneider , R. Gounder , Science 2017, 357, 898. 28818971 10.1126/science.aan5630

[119] C. Wang , Z. Liu , L. Wang , X. Dong , J. Zhang , G. Wang , S. Han , X. Meng , A. Zheng , F. S. Xiao , ACS Catal. 2018, 8, 474.

[120] C. I. Richards , S. Choi , J. C. Hsiang , Y. Antoku , T. Vosch , A. Bongiorno , Y. L. Tzeng , R. M. Dickson , J. Am. Chem. Soc. 2008, 130, 5038. 18345630 10.1021/ja8005644 PMC2766658

[121] L. A. Peyser , A. E. Vinson , A. P. Bartko , R. M. Dickson , Science 2001, 291, 103. 11141556 10.1126/science.291.5501.103

[122] J. Yu , S. A. Patel , R. M. Dickson , Angew. Chem., Int. Ed. 2007, 46, 2028. 10.1002/anie.200123456 PMC2754274 17285671

[123] G. Calzaferri , C. Leiggener , S. Glaus , D. Schürch , K. Kuge , Chem. Soc. Rev. 2003, 32, 29. 12596543 10.1039/b108571a

[124] G. De Cremer , B. F. Sels , J. Hotta , M. B. J. Roeffaers , E. Bartholomeeusen , E. Coutiño‐Gonzalez , V. Valtchev , D. E. De Vos , T. Vosch , J. Hofkens , Adv. Mater. 2010, 22, 957. 20217819 10.1002/adma.200902937

